# Cystamine Preparations Exhibit Anticoagulant Activity

**DOI:** 10.1371/journal.pone.0124448

**Published:** 2015-04-27

**Authors:** Maria M. Aleman, Lori A. Holle, Katherine G. Stember, Christa I. Devette, Dougald M. Monroe, Alisa S. Wolberg

**Affiliations:** 1 Department of Pathology and Laboratory Medicine, University of North Carolina at Chapel Hill, Chapel Hill, North Carolina, United States of America; 2 McAllister Heart Institute, University of North Carolina at Chapel Hill, Chapel Hill, North Carolina, United States of America; 3 Department of Medicine, University of North Carolina at Chapel Hill, Chapel Hill, North Carolina, United States of America; National Cerebral and Cardiovascular Center, JAPAN

## Abstract

Transglutaminases are a superfamily of isoenzymes found in cells and plasma. These enzymes catalyze the formation of ε-N-(γ-glutamyl)-lysyl crosslinks between proteins. Cystamine blocks transglutaminase activity and is used *in vitro* in human samples and *in vivo* in mice and rats in studies of coagulation, immune dysfunction, and inflammatory disease. These studies have suggested cystamine blocks fibrin crosslinking and has anti-inflammatory effects, implicating transglutaminase activity in the pathogenesis of several diseases. We measured the effects of cystamine on fibrin crosslinking, tissue factor-triggered plasma clot formation and thrombin generation, and coagulation factor enzymatic activity. At concentrations that blocked fibrin crosslinking, cystamine also inhibited plasma clot formation and reduced thrombin generation. Cystamine inhibited the amidolytic activity of coagulation factor XI and thrombin towards chromogenic substrates. These findings demonstrate that cystamine exhibits anticoagulant activity during coagulation. Given the close relationship between coagulation and inflammation, these findings suggest prior studies that used cystamine to implicate transglutaminase activity in disease pathogenesis warrant re-examination.

## Introduction

Transglutaminases are a superfamily of 9 isoenzymes found in cells (transglutaminases 1–7 and erythrocyte band 4.2) and plasma (factor XIII, FXIII) in humans and related species. Eight of these proteins produce inter- and intra-molecular ε-N-(γ-glutamyl)-lysyl bonds to cross-link proteins and protein complexes; a ninth protein, erythrocyte band 4.2, has similar domain organization but lacks a catalytic cysteine residue essential for the transglutaminase active site [1]. FXIII is found in both cellular and plasma compartments. Plasma FXIII is activated by thrombin in the presence of calcium. Activated FXIII (FXIIIa) catalyzes crosslinks between plasma proteins and the γ- and α-chains in fibrin and stabilizes the fibrin network against mechanical disruption and biochemical dissolution.

Cystamine is a pan-transglutaminase inhibitor that blocks transglutaminase activity, including that of coagulation factor XIII(a). Cystamine has been used to inhibit transglutaminase activity *in vitro* in samples from humans and related species [2–5]. Cystamine has also been used *in vivo* in mice, rats, and *Drosophila*, where it is administered via subcutaneous, intraperitoneal, and intravenous injection, as well as in drinking water and food [2–4,6–13]. Cystamine administration is associated with protection against H_2_O_2_-induced reactive oxygen formation [3], decreased levels of inflammatory mediators (e.g., tumor necrosis factor α and interleukin-6) and reduced pathology in models of cystic fibrosis [14], collagen-induced arthritis [11], ventricular hypertrophy [9], inflammatory bowel disease [10], and neurodegenerative disorders including Huntington’s disease [2–4,8,12,13]. By extension, these studies implicate transglutaminase activity in the pathophysiologic mechanisms that promote these diseases.

Herein, we report that cystamine preparations exhibit anticoagulant properties during plasma clot formation via anti-factor XIa and anti-thrombin activity. These findings suggest that the efficacy of cystamine in disease models may stem from its anti-transglutaminase and/or anticoagulant activities.

## Materials and Methods

### Materials

Cystamine dihydrochloride was from Wako Pure Chemical Industries (Osaka, Japan) or Sigma (St. Louis, MO, USA); results were similar with both preparations. T101 was from Zedira (Frankfurt, Germany). Normal, pooled plasma was prepared from healthy donors as described [15]. Anti-fibrinogen polyclonal antibody from rabbit was from Dako (Carpinteria, CA, USA). AlexaFluor 488-conjugated goat anti-rabbit IgG was from Jackson ImmunoResearch Laboratories.

### Fibrin crosslinking

Plasma clot formation was triggered by addition of lipidated tissue factor (1:12,000 dilution of Innovin, final) and re-calcification (10 mM CaCl_2_, final) in the presence of cystamine (1–100 mM, final). Final reaction volumes were 45 μL (85% plasma, final). After 1 hour, clots were reduced, dissolved, and boiled. Proteins were separated and identified by SDS-PAGE and western blotting, as described [16]. Band intensity was quantified by densitometry using Image J software version 1.48. γ-monomer bands were normalized to normal, pooled plasma; γ-γ dimers and high molecular weight (MW) crosslinked species were normalized to clots formed in the absence of inhibitor.

### Clot formation (turbidity assays)

Plasma clot formation was performed as above and monitored by turbidity at 405 nm in a SpectraMax Plus384 plate reader (Molecular Devices, Sunnyvale, CA, USA). The onset, rate of turbidity increase, and peak turbidity change were calculated as described [17].

### Thrombin generation assays

Plasma thrombin was measured by calibrated automated thrombography, as described [15,17]. Briefly, thrombin generation was triggered by addition of tissue factor, phospholipids, and CaCl_2_ (1 pM, 4 μM, and 16.7 mM, respectively, final), in the presence of cystamine (1–100 mM, final).

### Amidolytic assays

Factors IXa, Xa, VIIa, XIa, and thrombin amidolytic activity were assayed by incubating purified enzymes with cystamine (0.2–200 mM, in a 2-fold serial dilution) and measuring the cleavage rate of chromogenic substrates (Pefachrome FIXa, FXa, FVIIa, FXa, and TG, respectively [Enzyme Research Laboratories, South Bend, IN, USA]). The K_i_ was determined by first calculating the apparent *K*_*i*_ (*K*_*i*, *app*_) using the equation *v*(*V*_*0*_)^-1^ = 1-[*I*](*K*_*i*, *app*_+[*I*])^-1^. Since the inhibitor and substrate are in competition, the true *K*_*i*_ was then calculated using the equation *K*_*i*_ = *K*_*i*, *app*_ (*K*_*m*_([*S*]+*K*_*m*_)^-1^).

### Statistics

Descriptive statistics for clot formation and thrombin generation were summarized using means and standard deviations. Parameters were compared between groups using analysis of variance (ANOVA) in Kaleidagraph version 4.1.3 (Synergy Software, Reading, PA, USA). Parameters showing significant differences were then analyzed with Dunnett’s post hoc testing, using untreated plasma clots (0 mM cystamine or T101) as the index. *P*<0.05 was considered significant.

### Ethics statement

This study was approved by the University of North Carolina at Chapel Hill Institutional Review Board (Approval #01–1274). Human subjects gave written informed consent before participation.

## Results

### Cystamine inhibits fibrin formation in plasma

**Fig 1:**
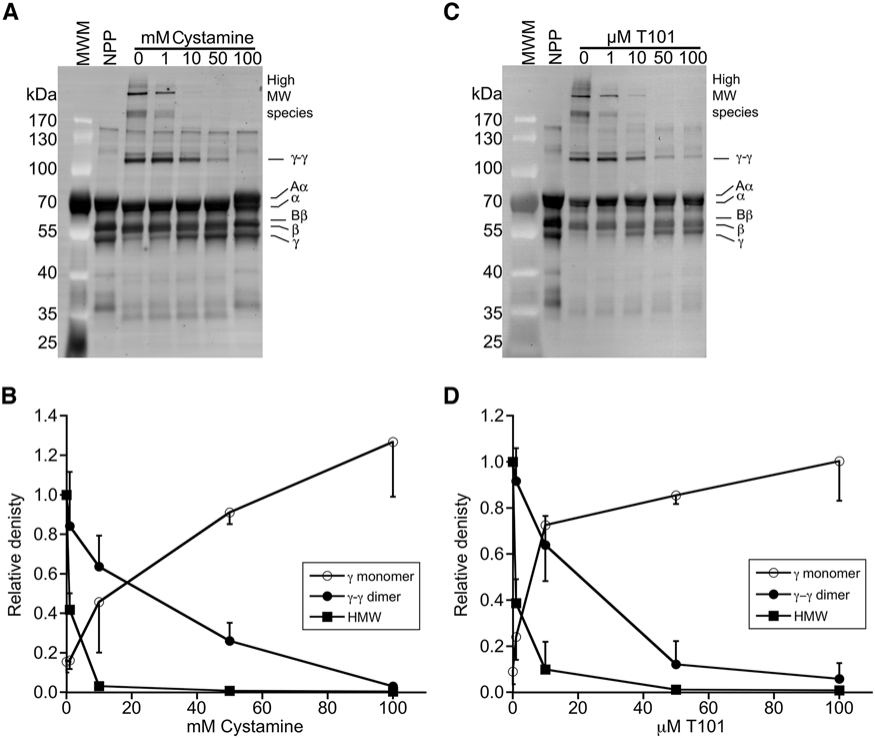
Cystamine inhibits fibrin crosslinking. Representative western blots and densitometry analysis showing the effects of cystamine (A, B) and T101 (C, D) on fibrin formation and crosslinking. Plasma was clotted by re-calcification and addition of lipidated tissue factor in the presence of cystamine or T101. Samples were reduced, boiled and separated by SDS-PAGE and identified by western blotting with anti-fibrinogen polyclonal antibody. MWM, molecular weight marker. NPP, un-clotted normal-pooled plasma. The upper high MW band (indicated by asterisk on the blot) was used for densitometry analysis of this species.

**Fig 2:**
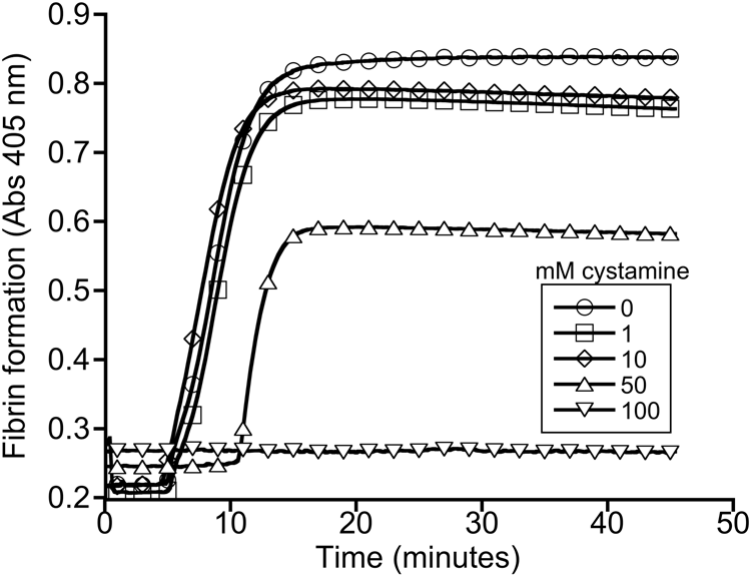
Cystamine inhibits plasma clot formation. Plasma clot formation was triggered by re-calcification and addition of lipidated tissue factor, in the presence of cystamine. Clot formation was monitored by turbidity. The data are representative of four experiments.

To identify a cystamine concentration that blocks FXIIIa transglutaminase activity, we added cystamine to plasma, triggered clotting by recalcification and addition of lipidated tissue factor, and analyzed fibrin crosslinking by SDS-PAGE and western blotting. Fig 1A and 1B show that cystamine caused a dose-dependent reduction in the formation of γ-γ dimers and high MW species, and 100 mM cystamine fully blocked fibrin crosslinking. Surprisingly, however, this concentration of cystamine also prevented fibrin formation, indicated by the lack of a shift of fibrinogen Aα and Bβ chains to fibrin α- and β-chains, respectively (Fig 1A). By comparison, 50 μM of the 2-[(2-oxopropyl)thio]imidazolium derivate transglutaminase inhibitor, T101, blocked γ-γ dimer formation without inhibiting fibrin formation (Fig 1C and 1D). Turbidity assays confirmed these findings; fifty mM cystamine significantly prolonged the lag time and reduced the turbidity change during clotting (Fig 2 and Table 1), indicating decreased fibrin formation. Consistent with the western blot analysis, 100 mM cystamine completely abrogated clot formation.

**Table 1:** Clot formation and thrombin generation parameters.

	mM cystamine				
	0	1	10	50	100
**Clot Formation**					
Onset (minutes)	4.6±1.5	4.8±1.4	5.4±2.2	10.0±2.8*	>60*
Rate (mOD/minute)	54.3±32.1	67.5±23.2	64.7±31.4	69.6±35.6	0*
Peak turbidity change	0.63±0.02	0.64±0.05	0.64±0.03	0.51±0.09*	0*
**Thrombin Generation**					
Lag time (minutes)	4.0±1.2	4.5±2.0	5.1±2.2	7.1±2.6	10.1±0.8*
Time to peak (minutes)	8.4±1.8	8.8±2.5	10.3±3.2	12.2±3.5	19.9±0.2*
Thrombin peak (nM)	97.3±38.0	62.2±25.9	18.8±9.2*	26.9±5.9*	5.1±1.8*
Endogenous thrombin potential (nM.minutes)	934.4±213.9	667.6±123.7*	277.3±108.1*	367.0±75.4*	107.3±28.6*

Mean ± standard deviation

*P<0.05 vs 0 mM cystamine (untreated plasma)

### Cystamine inhibits thrombin generation in plasma

**Fig 3:**
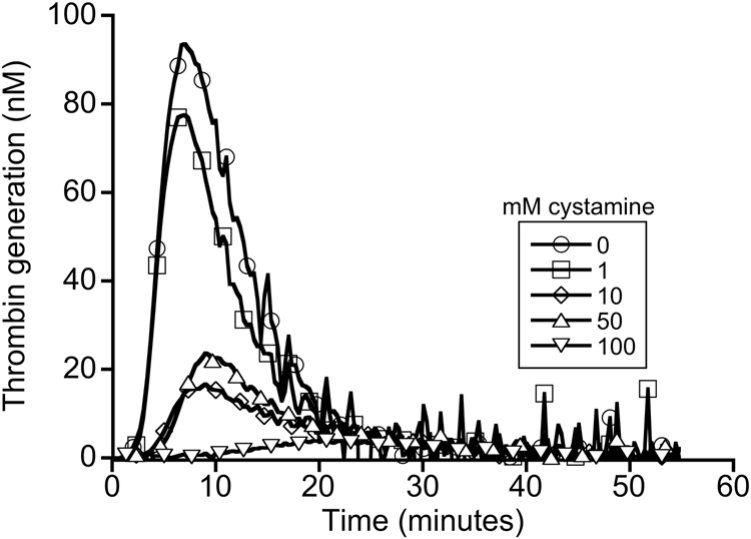
Cystamine inhibits thrombin generation. Plasma thrombin generation was triggered by re-calcification and addition of tissue factor and phospholipids, in the presence of cystamine. Thrombin generation was monitored by calibrated automated thrombography. The data are representative of four experiments.

During coagulation, thrombin mediates the proteolytic conversion of fibrinogen to fibrin. To determine whether cystamine inhibited fibrin formation by reducing thrombin generation, we measured plasma thrombin generation in the presence of cystamine. Consistent with the clotting data, we observed a cystamine concentration-dependent decrease in thrombin generation (Fig 3 and Table 1). Results were similar using cystamine preparations from two different companies. Effects of cystamine on thrombin generation appeared slightly more pronounced than its effects on fibrin formation, likely because even small amounts of thrombin are sufficient to convert fibrinogen to fibrin [18]. These findings suggest cystamine blocks one or more enzymes in the coagulation cascade that contribute to thrombin generation.

### Cystamine inhibits factor XIa and thrombin amidolytic activity

**Fig 4:**
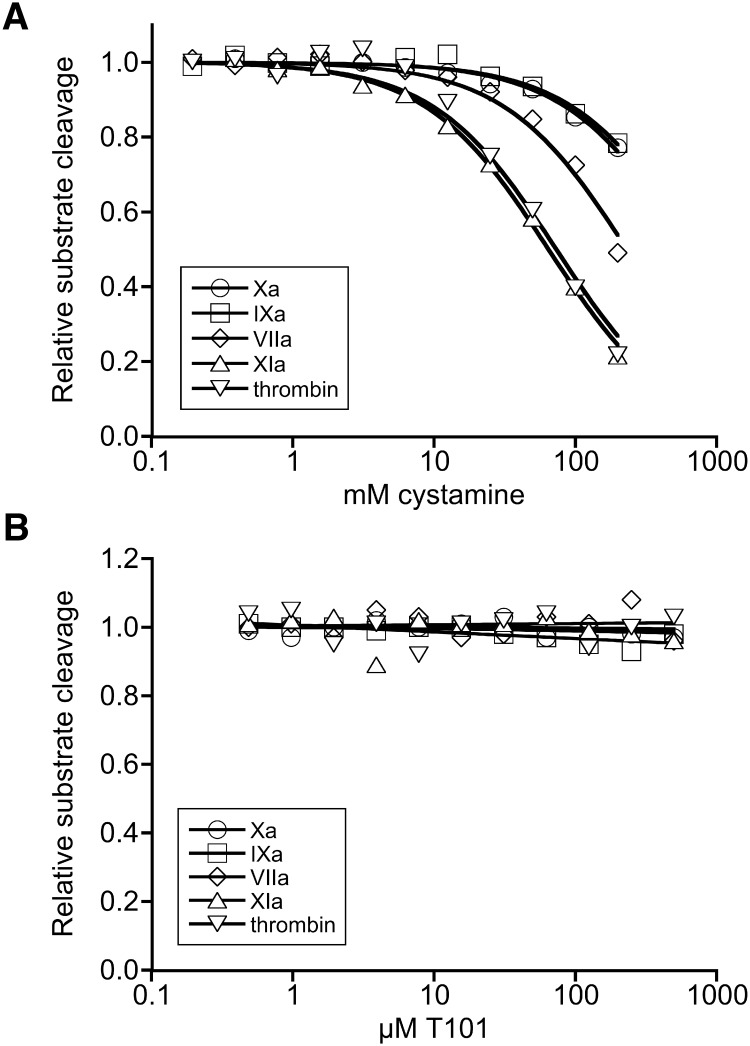
Cystamine inhibits the amidolytic activity of coagulation enzymes. Factors IXa (circles), Xa (squares), VIIa (diamonds), XIa (triangles), and thrombin (inverted triangles) amidolytic activity were assayed by incubating enzymes with cystamine (A) or T101 (B) and measuring the cleavage rate of chromogenic substrates. The data show means of 2–3 experiments per enzyme.

Coagulation involves a proteolytic cascade that culminates in the production of thrombin. To determine which enzyme in this cascade is inhibited by cystamine, we characterized the effect of cystamine toward key enzymes in the clotting pathway: factors VIIa, XIa, Xa, IXa, and thrombin. Cystamine only weakly inhibited factors VIIa, Xa, or IXa (*Ki* 180 mM, 108 mM, and 445 mM, respectively, Fig 4A). However, cystamine effectively inhibited both factor XIa and thrombin (*Ki* 41 mM and 54 mM, respectively). By contrast, T101 did not exhibit inhibitory activity against any of the enzymes tested (Fig 4B). Together, these data reveal that commercial cystamine preparations exhibit anticoagulant activity.

## Discussion

Transglutaminase activity is essential for a broad range of biological processes, including clot stabilization, atherosclerosis, and tissue regeneration, including skin barrier formation, extracellular matrix assembly, angiogenesis [19], and artery remodeling [20,21]. Accordingly, transglutaminase deletion or inhibition has pathologic effects, including increased bleeding, delayed wound healing, and pregnancy failure (reviewed in [22]). Interestingly, however, transglutaminase deletion or inhibition has also been shown to reduce the pathology in several disease models, including cystic fibrosis [14], collagen-induced arthritis [11], ventricular hypertrophy [9], inflammatory bowel disease [10], neurodegenerative disorders including Huntington’s disease [2–4,8,13], and venous thrombosis [16]. These findings suggest transglutaminase inhibitors could be a new approach for treating these disorders. Consequently, there is substantial interest in delineating these pathways using existing and new transglutaminase inhibitors in basic and preclinical studies.

Of the currently available transglutaminase inhibitors, cystamine is frequently used because it is inexpensive and easy to administer in both *in vitro* and *in vivo* models. Its effects have been used to define the contribution of transglutaminase activity in a range of disorders. As such, our finding that cystamine exhibits anticoagulant activity is important. Inflammation and coagulation pathways are often both activated in autoimmune and thrombotic disorders, and both transglutaminase and procoagulant activities have been implicated in several disease presentations, including rheumatoid arthritis [11,23,24], ventricular hypertrophy [9,25], and celiac disease [26,27]. Indeed, models of ventricular hypertrophy and rheumatoid arthritis have each shown protective effects of cystamine and anticoagulants [9,11,23–25]. Interestingly, in a model of Huntington’s disease, treatment with cystamine delays the onset of motor dysfunction and improves lifespan even in mice that do not express tissue transglutaminase [28], demonstrating anti-transglutaminase-independent effects of cystamine in a mouse model. The nature of these effects was not determined.

In summary, our data demonstrate anticoagulant activity in commercial cystamine preparations. Together with previous findings, these data suggest cystamine’s effects on *in vitro* and *in vivo* disease models may stem from its anti-transglutaminase activity, its anticoagulant activity, both, or even additional mechanisms of action. The current findings warrant renewed investigations into the operant mechanisms of this agent in disease.
